# Iodine Supplementation: Usage “with a Grain of Salt”

**DOI:** 10.1155/2015/312305

**Published:** 2015-03-19

**Authors:** Alessandro Prete, Rosa Maria Paragliola, Salvatore Maria Corsello

**Affiliations:** Endocrinology Unit, Università Cattolica del Sacro Cuore, Largo Gemelli 8, 00168 Rome, Italy

## Abstract

Iodine supplementation through salt iodization is a worldwide, effective strategy for preventing iodine deficiency-related problems. Its safety and efficacy profile has been extensively investigated, and benefits far outweigh the potential iodine-induced risks. Moreover, iodine supplementation during pregnancy in order to avoid brain damage in the newborn is considered a mainstay of preventive medicine. Exposure to high amounts of iodine is actually well tolerated in most cases and can be unrecognized. Nevertheless, at-risk individuals may develop thyroid dysfunction even when they are exposed to increases in iodine intake universally considered as safe. Iodine-induced thyroid disorders include thyroid autoimmunity, thyrotoxicosis, iodine-induced goiter, and hypothyroidism. Moreover, a relationship between iodine intake and histotype distribution of differentiated thyroid cancer has been observed, with a progressive shift from follicular to papillary thyroid cancer. To date, evaluating iodine status in a clinical setting has limitations, and assessing the actual risk for each individual can be challenging, since it is influenced by personal history, genetics, and environmental factors. In conclusion, iodine supplementation programs need to be continued and strengthened, but iodine should be used “with a grain of salt,” because a growing number of susceptible individuals will be exposed to the risk of developing iodine-induced thyroid disorders.

## 1. Introduction

Iodine deficiency is a significant public health problem and worldwide efforts have been made over the past 80 years in order to eliminate this condition. However many countries, including some developed nations, are still considered to be moderate to mild iodine-deficient and billions of people remain at risk for iodine deficiency all over the world. Iodine supplementation through salt iodization is an effective and sustainable strategy for preventing iodine deficiency [1, 2]. Many countries have adopted salt fortification programs and the most extreme manifestations of iodine deficiency are becoming increasingly rare. Nonetheless, iodine deficiency is still a major public health problem, with pregnant women, infants, and school-age children being the most vulnerable categories.

Iodine deficiency-related problems are well known (hypothyroidism, goiter, and hypothyroidism-related brain damage in the newborns), but iodine excess-related thyroid disorders can be a clinical problem as well.

Most people are highly tolerant to iodine and can be chronically exposed to massive amounts of iodine without developing apparent side effects [3].  Table 1 reports the recommended daily allowance (RDA) and the upper intake levels (ULs) for iodine proposed by two institutions: the Health & Consumer Protection Directorate-General of the European Commission [4] and the Food and Nutrition Board of the U.S. Institute of Medicine of the National Academies [5]. ULs are defined as the highest daily intake levels that are unlikely to cause health problems in most of the individuals.

The aim of this review is to assess potential risks deriving from both physiological iodine exposure and above-physiological iodine exposure and to identify at-risk groups who are susceptible to high iodine exposure in order to predict, prevent, early detect, and treat iodine-induced thyroid disorders.

## 2. Iodine Exposure and Potential Development of Thyroid Disease

Risk for thyroid disease deriving from iodine exposure is U-shaped: this indicates that potential harm for the subject can derive from both iodine deficiency and iodine excess [6]. This relationship and the actual risk for each individual are influenced by many variables: age, gender, genetic predisposition, environmental factors, personal history of thyroid diseases, concurring diseases, and some medications. Thus it can be difficult to assess the actual risk for every single person.

Natural and artificial iodine sources are many, and exposure to “more than adequate” or “excessive” iodine levels can occur quite unknowingly. Exposure to very high doses of iodine may cause significant thyroid dysfunction in susceptible individuals. This predisposition resides broadly in a dysfunction of the self-regulatory mechanisms of thyroid hormone biosynthesis, which protect the thyroid gland from the fluctuations of iodine intake and from iodine overload. However, thyroid dysfunction can occur in patients with no history of thyroid disease.

Iodine supplementation through salt iodization is universally considered as a safe prevention strategy. It aims at exposing populations to physiological iodine amounts, and the potential risk of causing “more than adequate” or “excessive” iodine intake is low. However, evidence exists about iodine-induced thyroid disorders in areas with long-standing iodine deficiency where salt iodization is introduced or increased according to controlled prevention national programs.

Rather new sources of iodine are dietary supplements (such as kelp), multivitamins, and over-the-counter (OCT) drugs. National regulations regarding these drugs may vary substantially, and the product labeling can be misleading. In fact, some food supplements [8], prenatal multivitamins [9], and OCT drugs [10] have been proven to contain very high doses of iodine (up to several milligrams of elemental iodine/day), and actual iodine content can be higher than the one reported by the product labeling. The American Thyroid Association (ATA) has recently released a statement advising against the ingestion of dietary supplements containing >500 *μ*g of iodine daily (considering an UL for iodine of 1,100 *μ*g/day) [11].

## 3. How to Assess Iodine Intake

It is very important to stress the difference between taking “adequate,” “more than adequate,” and “excessive” iodine. The World Health Organization (WHO) uses the median urinary iodine excretion (UIE) to define “excessive” iodine intake in the overall population (Table 2): median UIE > 300 *μ*g/L (for general population) and >500 *μ*g/L (for pregnant women) are considered “excessive” and should be avoided for the potential adverse effects on health.

Moreover, the WHO points out that median UIE > 200 *μ*g/L should be avoided in populations characterized by long-term iodine deficiency that are exposed to significant increase of iodine intake [7]. In particular, these populations may be at risk for developing iodine-induced hyperthyroidism, since the thyroid has become very efficient in taking up and processing even small amounts of iodine. Recent national surveys based on median UIE showed that the number of countries with “more than adequate” or even “excessive” iodine intake has increased over the past years, indicating that a growing number of susceptible individuals are exposed to the risk of developing iodine-induced thyroid dysfunction [1].

In common clinical practice, spot urinary iodine values are generally used to assess iodine status. Nonetheless, there is substantial diurnal and day-to-day variation in UIE, and less than ten urine samples in an individual may be misleading [12]. Therefore, UIE is more useful for groups and population studies than for individuals.

Thyroid volume can be used as another parameter to evaluate iodine status in the general population. It tends to decrease after months/years of iodine supplementation in previously iodine-deficient communities, as shown in school-age children in Côte d'Ivoire [13]. Thyroid size and goiter volume can be easily assessed by ultrasound according to international reference values [14], and goiter shrinkage can be considered a sensitive long-term indicator of iodine sufficiency. Anyway, to date thyroid volume cannot be used to assess “more than adequate” or “excessive” iodine intakes.

Serum thyroglobulin (Tg) has been proposed as an alternative biomarker to assess iodine intake. A rise in thyroid-stimulating hormone (TSH) caused by iodine insufficiency can be responsible, indeed, for enhanced Tg production and cleavage: this leads to increased serum Tg levels. 13 *μ*g/L has been proposed as a cut-off serum Tg level to distinguish between insufficient and adequate iodine intake, especially in school-age children [15]. Anyway, data regarding adults and pregnant women are conflicting and large national and international randomized placebo-controlled trials are needed to assess the role of serum Tg as a biomarker of iodine intake in the general population. To date Tg cannot be used to as a biomarker to establish “more than adequate” or “excessive” iodine intakes.

## 4. Iodine “Excess” and Risk of Thyroid Autoimmunity

**Figure 1:**
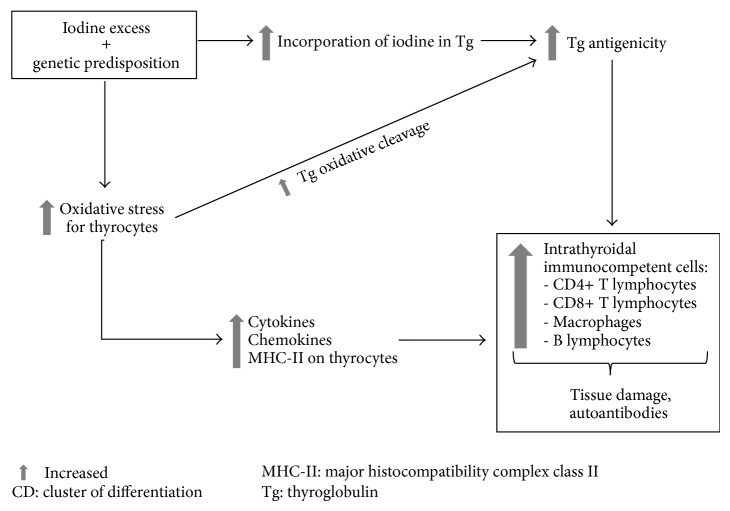
Iodine “excess” and thyroid autoimmunity. Iodine can damage thyrocytes directly, through intracellular oxidative stress, and indirectly, through activation of proinflammatory phenomena that recruit immunocompetent cells.

Iodine “excess” is an environmental risk factor for the development of thyroid autoimmunity, both in genetically prone animal models (such as the nonobese diabetic, NOD, mouse) and human population studies [16]. Iodine seems to stimulate thyroid autoimmune damage in a dose-dependent manner, and several mechanisms have been advocated (Figure 1).

Iodine excess can enhance oxidative stress of thyrocytes by activation of the reactive oxygen species (ROS) cascade, which is normally required for iodine oxidation and organification [17]. This is possibly caused by a latent alteration of intracellular processing of iodine, which is unmasked by iodine overload. This may lead to cellular injury (including apoptosis or necrosis) and proinflammatory changes in genetically prone thyroid tissue, such as increased cytokine and chemokine production [18, 19], major histocompatibility complex (MHC) class II expression [20], and intercellular adhesion molecule (ICAM)-1 expression [21] on thyrocytes. Moreover, excess iodine can change the 3D structure of Tg either by greater iodine organification [22] or by ROS-mediated Tg proteolysis [23]. As a consequence Tg autoantigenicity increases, leading to attraction of immunocompetent cells to the thyroid. All these changes are potential triggers for the development of thyroid autoimmunity, both as immune cell thyroid infiltration and autoantibody production. Both tissue infiltration and autoantibody production can be stopped by silencing CD4+ T lymphocyte, CD8+ T lymphocyte, or B lymphocyte response [24], indicating a pivotal role of lymphocytic infiltration in the development of iodine-induced autoimmune thyroiditis.

Several human population studies have shown a positive correlation between iodine exposure and the development of autoimmune thyroiditis. Li et al. [25] have shown an increased incidence of anti-Tg antibodies during a 5-year follow-up in a Chinese region with chronic excessive iodine exposure, and high iodine intake was a risk factor for developing hypothyroidism in antibody-positive subjects. Moreover, iodine prophylaxis has been put in relation to a higher incidence of Hashimoto's thyroiditis in Italy [26], antithyroid peroxidase (TPO) antibody positivity in Polish adults [27], and anti-Tg antibody positivity in female school-age children in Sri-Lanka [28]. A gradual increase of the incidence of Hashimoto's thyroiditis was also observed in Slovenia [29] after increase in salt iodization from 10 to 25 mg of potassium iodide per kg of salt in 1999. Finally, a study from Greece [30] shows how the shift from iodine insufficiency to adequate or excessive iodine intake has been accompanied by a higher rate of thyroid autoimmunity, mainly in young women. The prevalence of autoimmune thyroiditis in school-age children raised from 3.3% in 1994 to 9.6% in 2001, and the association between higher UIE and autoantibody positivity was significant.

## 5. Iodine “Excess” and Risk of Thyrotoxicosis

Excessive iodine intake may be responsible for iodine-induced thyrotoxicosis (Jod-Basedow phenomenon), caused by iodine action on thyrocytes whose function is TSH-independent. Therefore, susceptible subjects are patients with Graves' disease (even if in remission or under medical treatment) and patients with nodular goiter living in iodine-deficient areas. The first group usually includes younger subjects with diffuse goiters and detectable thyroid-stimulating antibodies (TSAb). On the other hand, the second group consists of older subjects with undetectable TSAb and nodular goiters which harbor areas of functional autonomy.

As a consequence, living in moderate to mild iodine-deficient areas is a risk factor* per se* for the development of thyrotoxicosis if the population is exposed to a sudden increase of iodine intake. The actual extent of this phenomenon depends on the duration of iodine deficiency, on the severity of iodine deficiency, and on the degree of iodine load [31]. By far the best documented epidemic of iodine-induced thyrotoxicosis occurred in Tasmania in the late 1960s and beyond [32], after the introduction of iodized bread and iodophors in the dairy industry and after increasing distribution of iodine-rich foods imported from Australia. Thyrotoxicosis was most evident in older subjects (>40 y.o.). Similar results have been observed in Austria after an increase in salt iodization in 1990 from 10 to 20 mg of potassium iodide per kg of salt [33]. An increase of cases of hyperthyroidism was observed, peaking 1–4 years after the enhancement of iodine prophylaxis: toxic nodular goiter was responsible for 75% of all cases, whereas Graves' disease accounted for 19%. The preferential incidence of iodine-induced thyrotoxicosis among older subjects can be responsible for a higher rate of complications, such as cardiac arrhythmias and heart failure.

However, the incidence of iodine-induced thyrotoxicosis tends to decrease over time after the introduction of iodine supplementation. In fact, prophylaxis is expected to reduce the number of patients with toxic nodular goiter, which are susceptible to the Jod-Basedow phenomenon.

## 6. Iodine “Excess” and Risk of Iodine-Induced Goiter and Hypothyroidism

A chronic exposure to iodine excess can lead to the development of goiter, with or without hypothyroidism. Rarely, hypothyroidism without goiter can occur.

**Figure 2:**
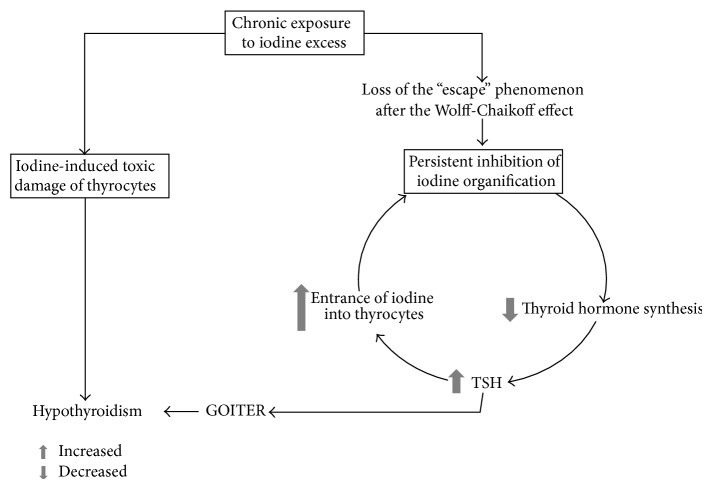
Iodine “excess,” goiter, and hypothyroidism. On the one hand, chronic exposure to iodine excess can cause direct oxidative stress of thyroid cells by activation of the ROS cascade. On the other hand, the loss of downregulation of NIS leads to persistent high intracellular levels of iodine that causes reduction in thyroid hormone production.

Two different pathogenic mechanisms have been advocated: direct, iodine-induced toxic damage of thyrocytes and loss of the “escape” phenomenon after the Wolff-Chaikoff effect, which is a physiological response likely due to downregulation of sodium-iodide symporter (NIS) (Figure 2).

Predisposing clinical conditions are Hashimoto's thyroiditis, previous treatment with radioactive iodine (especially for Graves' disease), history of external thyroid irradiation, previous subtotal thyroidectomy, postpartum and subacute thyroiditis, cystic fibrosis, iron overload (e.g., patients undergoing repeated blood transfusions and hemochromatosis), some drugs (e.g., interferon *α*, lithium, and phenazone). Fetuses and newborns (especially preterm infants) are at-risk categories* per se*, since the “escape” phenomenon after Wolff-Chaikoff effect fully develops in late gestation [34], and possibly because of the dose-dependent iodine storage by placenta [35]. Thereby neonatal goiter and congenital hypothyroidism can occur in case of maternal excessive iodine intake [36, 37]. Moreover, cases of acquired neonatal hypothyroidism due to iodine overload have been observed in preterm infants [38], full-term infants with congenital heart disease [39], and infants exposed to iodine-containing antiseptics during delivery [40] and during breastfeeding [41].

Pregnancy and breastfeeding are conditions requiring increased need for iodine, in order to ensure normal development of the brain and nervous system of the fetus and the baby. During pregnancy and lactation it can be necessary to introduce oral supplements to cover the daily requirement of 220–290 *μ*g iodine/day. The ATA recommends the assumption of oral supplements containing 150 *μ*g/day of iodine (in the form of potassium iodide) in North American women during pregnancy and lactation. At the same time, however, the ATA does not recommend routine daily intake of an amount of iodine >500 *μ*g/day (according to the WHO) or >1,100 *μ*g/day (according to the U.S. Institute of Medicine), because of the potential risk of fetal hypothyroidism [42].

## 7. Iodine “Excess” and Thyroid Cancer

Thyroid cancer incidence has increased dramatically worldwide during the past decades. This “epidemic” may be apparent and only related to improved diagnostic techniques, as suggested by several authors [43]. However, a potential role of increasing iodine supplementation in this phenomenon has been advocated [44].

Iodine intake in populations seems to affect mostly histotype distribution of differentiated thyroid cancer. In 1985 Williams [45] observed different papillary (PTC) to follicular (FTC) thyroid cancer ratios in several areas, according to changes in iodine intake. Iodine deficiency was associated with a PTC/FTC ratio of 0.19–1.7. This ratio was 1.6–3.7 in areas with moderate iodine intake, while it rose to 3.4–6.5 in regions with high iodine intake. Hence, it is possible that introducing iodine supplementation in iodine-deficient areas may lead to an increased PTC/FTC ratio [46, 47]. However, iodine intake does not seem to affect the overall incidence of thyroid cancer in populations [48].

The shift from FTC to PTC may be related to a higher rate of V600E BRAF mutation over time [49]. A possible role of increased iodine intake in affecting the genetics of thyroid cancer, such as an oxidative stress-induced DNA damage, has been advocated but extensive data are lacking. Guan et al. [50], however, showed that PTC harboring V600E BRAF mutation are more frequent in Chinese areas with a high iodine intake when compared to control areas. Moreover, the authors observed that BRAF-positive PTC are associated with a worse anatomopathological and clinical outcome.

## 8. Iodine Status and TSH: Should We Rethink TSH Reference Range?

Iodine status influences TSH levels. Adequate iodine intake is associated with smaller thyroid volume in healthy individuals: hence higher TSH levels may be required as a compensatory mechanism to ensure physiological thyroid hormone production. Moreover, thyrocyte response to TSH is influenced by iodine status, with higher iodine intake triggering decreased sensitivity of thyroid to TSH. This is in line with Studer's hypothesis regarding natural heterogeneity of thyrocytes [51].

Several studies show that shift to an adequate iodine intake is associated with a trend of higher TSH levels [52, 53]. Whether these data reflect only a physiological adaptation or suggest an underlying increased risk for hypothyroidism is still debated. For example, epidemiological data from Denmark (a previously iodine-deficient country) show a progressive shift of thyroid disease from hyperthyroidism in the elderly [54] to thyroid autoimmunity [52], in line with increased iodine fortification. However, recent data about German children and adolescents with no history of thyroid disease [55] show that higher UIE are associated with higher TSH levels (within the reference range) and lower thyroid volume. Reassuringly, higher TSH levels did not show correlation with anti-TPO antibody positivity.

Therefore the ongoing improvement of iodine status worldwide through salt iodization is likely to influence the homeostatic set point of the hypothalamus-pituitary-thyroid axis in an increasing number of previously iodine-deficient populations. We will probably have to rethink TSH reference range according to changes in iodine status, and this may affect current approaches to clinical settings such as pregnancy and congenital screening for hypothyroidism, and to some thyroid disorders, such as subclinical hypothyroidism.

## 9. Conclusions

Salt iodization stands out as the most safe and effective way of improving iodine status in the general population. It is a mainstay of preventive medicine and extensive data support its fundamental role in avoiding iodine deficiency-related problems. Salt iodization is part of a broader discourse about thyroid and micronutrients such as selenium, whose role in thyroid physiology and disease is under continuous investigation [56].

There are no known benefits deriving from taking doses of iodine above the RDA and its “excessive” assumption should be discouraged, since adverse health effects may develop. In the evaluation of a patient with thyroid dysfunction it is mandatory to investigate the possible assumption of dietary supplements and OTC drugs containing iodine and the potential exposure to other exogenous sources rich in iodine (kelp and other seaweeds; some drugs, such as amiodarone; iodinated radiocontrast agents; some foods).

It is important to highlight the need for national and international surveillance programs of population iodine intake: constant and ongoing investigation is needed in order to avoid potential harms deriving from exposing susceptible individuals to “more than adequate” or “excessive” iodine intake. Particular attention should be paid to individuals at risk for developing iodine-induced thyroid disorders: the elderly, fetuses, and the newborns are the most vulnerable groups.

Countries who adopt salt fortification have developed national surveillance strategies and international surveys are regularly carried out to assess iodine status worldwide. Most of the data come from random school-age children spot urinary iodine values: as stated above, this approach has limitations. Large randomized placebo-controlled trials are needed to update age- and pregnancy-specific UIE cut-off levels and to possibly investigate newer biomarkers of iodine intake, such as Tg serum levels. Cautious control of the degree of salt fortification, iodized salt distribution, and salt consumption should be met.

In this context, the aims of both dietary salt reduction (to decrease cardiovascular risk) and adequate iodine intake (to decrease iodine deficiency consequences) could not be in contrast. For example, a recent Italian study shows that a recommended salt intake of 5 g/day, if iodized at 30 mg/kg, is sufficient to guarantee adequate iodine intake both in adults and children [57].

Monitoring of prescription rates of thioamides can be a further tool to detect iodine-induced hyperthyroidism in the general population. Finally, public control should be extended not only to salt iodization, but also to iodine-containing medications, dietary supplements, and chemicals used in farming and the food industry. Health education projects in order to teach general population the benefits (many) and the potential risks (few) of iodine exposure can be a valuable option as well. These are effective strategies, aimed at exposing the general population to an optimal iodine intake. Social and health costs can therefore be reduced by preventing new cases of thyroid disorders.

## Figures and Tables

**Table 1:** RDA and ULs for iodine according to the European Commission [4] and to the U.S. Institute of Medicine of the National Academies [5].

Age	RDA (*μ*g/day)	ULs for iodine (*μ*g/day)	
		Health & Consumer Protection Directorate-General (European Commission)	Food and Nutrition Board (U.S. Institute of Medicine of the National Academies)
0–12 months	Not established^*^	Not established^*^	Not established^*^
1–3 years	90	200	200
4–6 years	90	250	300
7-8 years	90	300	300
9-10 years	120	300	600
11–13 years	120	450	600
14 years	150	450	900
15–17 years	150	500	900
Adults	150	600	1,100
Pregnant women	220	600	14–18 years: 900 19+ years: 1,100
Breastfeeding women	290	600	14–18 years: 900 19+ years: 1,100

^*^Food and formula milk should be the only sources of iodine in this age group.

RDA: recommended daily allowance; UL: tolerable upper intake level.

**Table 2:** Median UIE and iodine intake according to the WHO [7].

Median UIE	Iodine intake
General population	
<99 *μ*g/L	Insufficient
100–199 *μ*g/L	Adequate
200–299 *μ*g/L	More than adequate
≥300 *μ*g/L	Excessive

Pregnant women	
<150 *μ*g/L	Insufficient
150–249 *μ*g/L	Adequate
250–499 *μ*g/L	More than adequate
≥500 *μ*g/L	Excessive

Breastfeeding women and children younger than 2 years old	
<100 *μ*g/L	Insufficient
≥100 *μ*g/L	Adequate

UIE: urinary iodine excretion.
